# Identifying Highly Connected Counties Compensates for Resource Limitations when Evaluating National Spread of an Invasive Pathogen

**DOI:** 10.1371/journal.pone.0037793

**Published:** 2012-06-12

**Authors:** Sweta Sutrave, Caterina Scoglio, Scott A. Isard, J. M. Shawn Hutchinson, Karen A. Garrett

**Affiliations:** 1 Department of Plant Pathology, Kansas State University, Manhattan, Kansas, United States of America; 2 Department of Electrical and Computer Engineering, Kansas State University, Manhattan, Kansas, United States of America; 3 Departments of Plant Pathology and Meteorology, Pennsylvania State University, University Park, Pennsylvania, United States of America; 4 Department of Geography, Kansas State University, Manhattan, Kansas, United States of America; INSERM & Universite Pierre et Marie Curie, France

## Abstract

Surveying invasive species can be highly resource intensive, yet near-real-time evaluations of invasion progress are important resources for management planning. In the case of the soybean rust invasion of the United States, a linked monitoring, prediction, and communication network saved U.S. soybean growers approximately $200 M/yr. Modeling of future movement of the pathogen (*Phakopsora pachyrhizi*) was based on data about current disease locations from an extensive network of sentinel plots. We developed a dynamic network model for U.S. soybean rust epidemics, with counties as nodes and link weights a function of host hectarage and wind speed and direction. We used the network model to compare four strategies for selecting an optimal subset of sentinel plots, listed here in order of increasing performance: random selection, zonal selection (based on more heavily weighting regions nearer the south, where the pathogen overwinters), frequency-based selection (based on how frequently the county had been infected in the past), and frequency-based selection weighted by the node strength of the sentinel plot in the network model. When dynamic network properties such as node strength are characterized for invasive species, this information can be used to reduce the resources necessary to survey and predict invasion progress.

## Introduction

Invasive species are a global problem in natural, agricultural, and human health systems, and there is a corresponding great need for optimized strategies for detection of invasive species movement [1]. For human pathogens, it may be possible to integrate standard records collected in clinics in an analysis of spatial spread. For many invasive species, however, time-consuming field surveys performed by trained personnel are needed to track invasions. Despite the cost, information about invasive spread is critical for managers at many levels, from policy makers to individual land managers. The trend toward large research networks, such as the US National Ecological Observatory Network (NEON), is another motivation for identifying optimal approaches to sampling across large linked ecological systems [2]. Sampling strategies and strategies for identifying points for management of invasive species will often have significant overlap. The spatial structure of landscapes to be sampled is an important consideration in constructing ecological sampling designs and measures of invasion [3], [4]. Taking into account the dispersal mechanisms of invasive species in combination with the connectivity of the landscape for invasion has the potential to improve management efforts [5], [6]. For example, particular natural or artificial water bodies may function as invasion hubs, so that management of invasives in those lakes may have greater impact [7], [8]. Human transport hubs may also have important roles in invasions [9] and models of vaccination programs in human and other animal populations may often be relevant to invasions in plant landscapes [10], [11], [12], [13], [14].

The rapid development of network modeling approaches promises new insights into spatial and temporal components of biological invasions. In typical applications of network models to spatial processes, locations or individuals are ‘nodes’ and the probability of encounters or movement between any two nodes is described by the weight of the ‘link’ that joins the two nodes. Network model applications in biology have included movement of animals among habitat patches and movement of pathogens through a host population [15]. The connectivity of a network plays an important role in determining the dynamics of epidemics [16].

Recently, network models have been explored for a range of plant epidemics [17], [18], [19], [20], [21], [22], [23]. For example, Margosian et al. [24] considered a network of US counties, with links between adjacent counties weighted as a function of crop densities, and analyzed the connectivity of the landscape based on different threshold density levels. However, this model was limited to identifying more and less strongly connected regions and did not directly predict the course of an invasion process. Balcan et al. [25] is an example of national and global scale modeling of human epidemics based on human movement, but there is a need for a network modeling platform for large-scale landscapes outside human transportation networks.

The prediction of the progress of an epidemic can be mathematically represented using many different models. In SEIR models, individuals in the population are categorized as Susceptible (S), Exposed (E), Infected (I), or Recovered (R). Depending on the selected subset of possible compartments, SI, SIR, SEIR, or SIS models can be considered, which can be appropriate for different infectious processes. A set of differential equations can be written to represent the change in the fraction of the population in each compartment over time. In a SIR model, the existence of a threshold for the aggressiveness of the infection above which a major outbreak will occur was proved by Kermack and McKendric in 1927 [26]. When the population is not very large, deterministic models are not accurate enough, so probabilistic models were proposed where a set of differential equations can be written to represent the change in the probability that a single node is in a given compartment. For some probabilistic models, an epidemic threshold can be defined [27]. When the homogeneity assumption is not realistic, a further refinement can be obtained by the use of the concept of a contact network; a given node is only in contact with the subset of nodes to which it is linked. Using a network to represent the contacts among nodes, model predictions can become more accurate at the expense of increased complexity. Furthermore, depending on the scale of the representation of the population, nodes can represent not only individuals but also groups of individuals. In the latter case the models are called meta-population schemes and include information about the movements of subpopulations between nodes.

A network is characterized by an adjacency matrix (a matrix of link weights or indicators for the presence or absence of a link between two nodes) and by features such as the node degree (the number of links connected to a given node), shortest path (the shortest distance between any given pair of nodes), and the diameter (the longest shortest path). For all the network-based models, the topology of the network plays a key role in determining the characteristics of the spreading process [28]. Considering for example scale free networks, networks where the degree distribution follows a power law, Pastor-Satorras and Vespignani [29], [30] proved the absence of the epidemic threshold. In other words, all the infections can cause epidemics in infinite scale free networks, and for this reason such networks are very vulnerable. Conversely, regular networks, with almost constant node degree, and networks with a large diameter show robustness with respect to virus spread [31]. For general networks, Van Mieghem et al. [32] and Wang et al. [33] proved that the epidemic threshold is proportional to the inverse of the largest eigenvalue of the adjacency matrix. The model proposed by Wang et al. [33], and further extended by Schumm et al. [34] to weighted networks, serves as the basis for our model development below.

The structure of network models uses information about the status of individual nodes in an explicit way, suggesting their potential for analysis of sampling strategies. Strategies for identifying nodes in epidemic networks that are particularly important for vaccination efforts have been the subject of several studies (e.g., [10], [11], [12], [13], [14]). And in the context of computer network analyses, strategies for sampling the most important links have been evaluated (e.g., [35]). However, strategies for sampling invasive species in the resource-intensive context we have discussed here represent additional challenges [36], [37], [38], [39], [40], [41], [42], [43], [44], [45]. In this study, we present what is, to our knowledge, the first continental-scale test of the utility of network metrics for optimizing sampling strategies under limited resources, based on one of the most extensive data sets for an invasive species. The soybean sentinel plot network has been held up as an example of what can be accomplished in integrated national research programs such as the US National Ecological Observatory Network (NEON) [46]. The sentinel plot dataset is also an important resource for evaluating optimal sampling approaches for use in programs such as NEON.

Our first objective was to develop a dynamic network model for soybean rust in the USA, using the sentinel plot dataset and local information about host availability and wind speed and direction. We went a step beyond the model of Margosian et al. [24], by estimating parameters to describe the probability of movement between county nodes based on a subset of the sentinel plot dataset and by validating the model using another subset. Unlike the models of Balcan et al. [25], our model was based on probabilities of invasive species dispersal outside human transportation networks. Our second objective was to use this model to evaluate a set of strategies for sampling invasive movement under increasing limits on sampling resources. Beginning with the complete sentinel plot network, we evaluated the error in predictions when a smaller subset of the sentinel plots was retained following each of the following four strategies: (i) random selection of the subset, (ii) weighted probability of inclusion based on geographic zone, (iii) selection of the subset based on historical frequency of infection, and (iv) selection based on frequency of infection weighted by node strength (where the node strength of a given node is the sum of all link weights linked to that node, so that higher node strength indicates a node that is more ‘connected’).

One of the most intensely studied invasions has been that of soybean rust in the USA, an invasion of interest for several reasons. First, this invasive pathogen, *Phakopsora pachyrhizi*, is of great economic importance to soybean production. The disease had already caused great losses in other countries [47]. For example, in Brazil huge losses were reported in 2003 [48]. *P. pachyrhizi* can also infect over 95 other leguminous species [49], so its impact on native legumes is yet to be fully appreciated. Second, the ecology of the pathogen in the US allows for the study of ‘replicate’ invasions. The disease overwinters in the southeastern US, where the weed kudzu acts as a reservoir, and migrates annually to the north by windblown spores [50]. Third, the data set assembled by the impressive team studying soybean rust is one of the most substantial available for the study of invasive species. Significant efforts in modeling of soybean rust began about two decades ago [51] and the disease was detected in the continental United States in 2004 [52]. A network of hundreds of sentinel plots was organized by soybean researchers and organizations [53], as discussed in more detail below. An integrated aerobiological modeling system (IAMS) was developed to use the sentinel plot data for predicting progress of soybean rust, as described in Isard et al. [54], [55], [56]. For soybean rust, a critical decision for farmers is whether and when to apply a fungicide. If a fungicide is applied too early or in areas that will not be reached by the pathogen, the fungicide is wasted; if fungicide is not applied when needed, soybean yields will be substantially reduced [57]. Estimates of savings to U.S. soybean growers that have resulted from the extensive monitoring system in combination with the IAMS vary greatly [53], [57] and include a conservative calculation of ca. $200 million per year [58].

## Methods

### Invasion/Epidemic Model

As an overview, we developed a weighted dynamic network model in which the weights on the links were based on wind speed and direction and host density, which are driving factors for the spread of aerially dispersed pathogens. The centroid of each county in the US was considered to be a node or vertex. Some counties, most commonly in the southeastern US, contained sentinel plots, and thus functioned as ‘informative nodes’. The model presented here is a SI model within seasons. Also taken into account was the presence of the introduced weed kudzu (*Pueraria montana*) which acts as a reservoir for the pathogen and results in faster movement of the disease in regions where the weed is abundant. The rust status data collected from the soybean sentinel plots in the United States formed the basis for the construction and validation of the prediction model. The weight parameters were selected based on the construction data sets such that they gave reduced errors over the construction datasets. The selected weights were then applied to predict the progress of the disease for the validation datasets. The model was then used to compare four strategies for predicting epidemic spread when the number of informative nodes was systematically reduced. The strategy that performed best allowed good epidemic predictions for our observation years when the number of sentinel plots was reduced from approximately 500 to approximately 12.

#### Computing link weights and infection probabilities

Within a year, we used an SI model with nodes classified as being susceptible or infected. We considered the pathogen as spreading in a directed graph *G*(*N*, *L*) where *N* is the set of nodes and *L* is the set of links, and observed the system in discrete time steps 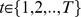. We assumed the pathogen moves across crop fields mainly by wind. We incorporated static as well as dynamic features of the network into the model, the approximately static component within a year being the host density (soybean crop density and kudzu weed density in our case study) and the dynamic component, including factors such as wind conditions which can be different at each time step. We modeled the link-weights as a function of these components where the weights varied at each time step (Table 1).

**Table 1:** Symbols used in the network model of pathogen invasion.

Symbol	Definition
*β_ij_*(*t*)	Wind-based component of infection rate between nodes *i* and *j* at time *t*
*ω_ij_*	Density- and distance-based component of infection rate between nodes *i* and *j* at time *t*
*u_ij_*(*t*)	Link-weight based on distance, density and wind between nodes *i* and *j* at time *t*
*p_i_*(*t*)	Probability that node *i* is infected at time *t*
*ζ_i_*(*t*)	Probability that node *i* will not receive infection from its neighbors at time *t*
*l_ij_*	Distance vector between nodes *i* and *j*
*w_i_*(*t*)	Wind vector at time *t* at node *i*

The key parameters of the link model are *ω_ij_* and *β_ij_*(*t*) which are combined into a single parameter *u_ij_*(*t*) that signifies the link-weight. Here, *ω_ij_* is a function of the parameters that are considered constant during the season: distance between nodes, and total crop density. In our first model, *ω_ij_* has a linear relationship with density and decays exponentially with distance. The exponential model and the power law model have frequently been used to model dispersal of plant pathogens over shorter distances [59], [60]. Equation 1 gives this exponential model where density is incorporated as a product, following a gravity law model of density effects [61].
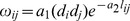
(1)


Here *d_i_* is the proportion host density (area of host/total area) in node *i*, *l_ij_* is the distance between nodes *i* and *j*, and *a*_1_ and *a*_2_ are two parameters. Two potentially useful variations on this model could be considered. One would include variations on exponential or power law models for dispersal. Another more general version of this model would incorporate two more parameters, such that *d_i_* and *d_j_* are taken to different powers reflecting the potentially different importance of source and destination node densities. These two parameters and potentially a parameterization allowing selection between exponential and power law models could be useful additions when there is sufficiently extensive data available at a large scale to estimate the parameters well.

The effect of an environmental variable such as wind can be incorporated in the model by considering infection rate *β_ij_*(*t*) as a function of wind speed and direction, set to be proportional to the scalar projection 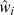
(*t*) of the wind in the direction of the link between the two nodes *i* and *j* (equation 2). Relevant functions might include the maximum wind speed for a given direction or average wind speed.
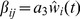
(2)


We combined *ω_ij_* and *β_ij_*(*t*) into a single parameter *u_ij_*(*t*) representing the link-weight as in the following equation 3.
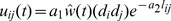
(3)


Many other different types of interactions among the distance, host density and wind could be considered. In a variation of the link model (equation 4), we considered another model for comparison in which the host density at source and destination nodes was added:
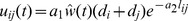
(4)


This might be appropriate in some cases, if the distribution of densities between source and destination nodes is not important, and fitting this second model allows us to evaluate the improvement in fit through use of the product of the densities.

Describing now the epidemic model on the weighted network, the probability of a node becoming infected depends on the number of infected neighbors. Under a mean field approximation, the probability *ζ_i_*(*t*) of a node *v_i_* not receiving infection from its neighbors is
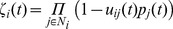
(5)where *p_j_*(*t*) is the probability of node *j* being infected at time *t*, link weights 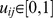
represent the level of contact, and *N_i_* is the set of neighboring nodes of node *i*. As a consequence, the probability *p_i_*(*t*) of node *i* being infected at time *t* is expressed as in equation 6.



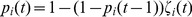
(6)Over time we updated the value of *β_ij_*(*t*) as a function of the wind data, and computed the probability of infection of each node at each time step.

#### Error definition

For the observed dataset, a value of 1 is assigned to the nodes which are observed as being infected and a value of 0 is assigned to the nodes which are observed not infected at time *t*. The simulation generates the predicted probabilities for infection of each node in the next time step. We define the error as the absolute difference between the data, considered as 1 if the node is infected or 0 if the node is healthy, and the correspondent infection probability computed by the model at the same time. Considering the errors related to infected nodes and the errors related to healthy nodes separately, the total error *E_in_*(*t*) in observed-infected nodes for time *t* can be computed as
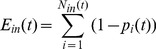
(7)where *N_in_*(*t*) is the total number of infected nodes at time step *t*. Similarly, the total error in observed-healthy nodes *E_hn_*(*t*) for the time-step *t* can be computed as
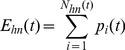
(8)where Nhn(t) is the total number of healthy nodes at time step t.

The average error can be calculated for comparison across time steps where sampling effort may vary. The mean error in infected nodes for time step *t* can be computed as
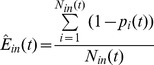
(9)and the mean error in healthy nodes for time step *t* can be computed as



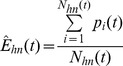
(10)Finally, the total error is obtained using a convex sum of error in the observed-infected nodes and the error in observed-healthy nodes
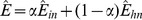
(11)where *α* is selected to assign different weights to errors in observed-infected nodes and errors in observed-healthy nodes.

#### Strategic reduction of invasion monitoring nodes

Once a model has been created, strategies can be developed to reduce the number of informative nodes necessary for predicting invasive movement accurately. One criterion for selecting a subset of nodes is to maximize the information obtained about the whole system. This can be performed through maximum entropy sampling. Unfortunately, this problem has been proven to be NP-Hard, such that the complexity grows rapidly with the size of the network [62]. Therefore, heuristic approaches are necessary to solve the problem.

When only a limited number of nodes can be sampled, there are several potential methods for selecting the most useful nodes. Here we discuss four methods in increasing order of information needed for implementation. Random selection of nodes is one approach, but does not make use of any information about the system. It may be used as a reference or ‘control’ method for determining the improvements in sampling performance provided by other methods. A second candidate approach is to sample progressively more heavily in zones where there is an a priori reason to expect that disease is more likely to occur. A third candidate approach is to select nodes that have been observed to be invaded more frequently in the past. A fourth candidate approach is to use information about the network traits, themselves, where a network trait such as node strength might be used as a measure for selecting nodes for sampling.

### The Case of Soybean Rust in the United States

#### Data

**Figure 1:**
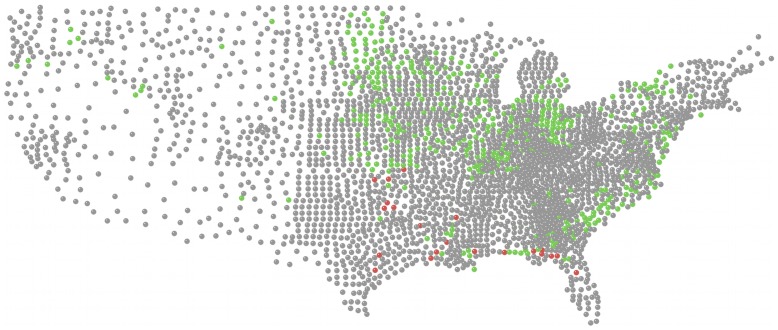
Observed soybean rust status in the United States in August 2007. Red nodes represent counties where infection was observed at least once during the time period, green nodes represent counties where no infection was found in sampling during the time period, and grey nodes represent counties where no observation was made during the time period.

We used the most extensive and coordinated data set currently available for any plant disease, and an exceptional data set for any invasive species, which illustrates the potential for NEON and similar megaprojects. The data from the US network of soybean sentinel plots from the years 2005 to 2008, publicly available from the ipmPIPE website (www.ipmpipe.org), was used to fit and validate our model. Soybean rust was first detected in the continental U.S. in early November 2004 after the soybean harvest. The sentinel plot monitoring system was established prior to the onset of the subsequent growing season in 2005. The rust dataset for each of the years from 2005 to 2008 was comprised of rust status (whether infection was found or not) for a given sentinel plot and the date of observation. The majority of sampling was done on a weekly to biweekly basis. Two soybean cultivars, one from the maturity group typically used in the surrounding area and the second from an earlier maturing cultivar group were generally planted on dates 1–2 weeks earlier than those in the surrounding commercial soybean fields. Scouting generally occurred on a weekly basis once the soybean plants began to flower and continued until they began to senesce. Prior to flowering scouting was usually less frequent. The sentinel plot network focuses on eastern counties (Fig. 1). Infection has been concentrated in the Southeastern United States, in part because of the presence of the weed kudzu in this region and the potential for overwintering in this perennial host.

The sentinel plot dataset did not include data from all counties at each time interval. There are many ways to estimate missing data in a map, with different types of assumptions and levels of complexity and accuracy. We estimated the soybean rust status for counties without observations using a simple but effective approach, as follows. If a node had previously been infected that season but had no new observations, it was assumed to continue to be infected. Our rationale was that once a county had been infected, even if soybean rust was no longer found in the sentinel plot it was likely to be present somewhere in the county. For nodes that had not been infected that year, we identified any other observed nodes within a distance d, increasing d from 0.5 degrees as necessary to encounter an observed node, and replaced the missing value with the mode of the neighboring nodes.

The soybean abundance data for the years 2005 to 2008 were accessed from the US National Agricultural Statistics Service (http://www.nass.usda.gov/Data and Statistics/index.asp). The data included for each year and county the FIPS identification number unique to a county, and the corresponding soybean area in acres. The soybean density was computed by normalizing the soybean acreage with the total county acreage. We also used kudzu abundance data with the corresponding kudzu area by county, using a data set compiled by Darryl Jewett in 2000 in which approximately 2 million acres of kudzu were reported. The density of kudzu was obtained by normalizing the kudzu acreage with the total county acreage. Wind data from first order weather stations were obtained from the National Climatic Data Center’s website. These data included the daily average resultant wind speed and wind direction for US first order weather stations, generally based at airports. Because first order weather stations are limited in number, we used the average wind velocity in space and time for each state.

#### Soybean rust model

There were approximately 500 informative nodes at each time step. We worked with one month time steps beginning in May and ending in September, the periods where data were generally available, giving four time steps per year and a total of 16 time steps across all the years.

We used the weight *u_ij_*(*t*) as in equations 3 and 4, where *ω_ij_* here is a function of the parameters which are constant during the season, such as distance between the nodes, and soybean density and kudzu density added together, to provide a total host density. In the case of soybean density, we considered a constant level during the growing season. We used a linear relationship between the wind-based infection rate, *β_ij_*(*t*), and the wind as a specific case for equation 2,
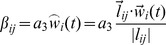
(12)where 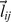 is the distance vector between the two nodes *i* and *j*, 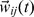 is the wind vector at a time *t* and 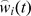 is the nonzero scalar projection of the wind vector at time *t* in the direction of the link between the two nodes, which was normalized by 13 mph (a speed determined based on the maximum monthly-average wind speed observed in any county during any month of the years considered).

We estimated parameters for the two models: a multiplicative model with gravity law for host densities (eq. 3), and a multiplicative model with sum of host densities (eq.4) based on the 2007 construction data set and validated the model with the 2005, 2006, and 2008 data sets. We used equations 5 and 6 to compute the *p_i_* for each time step. The parameters in the model were estimated by evaluating model fit for a grid of parameter values representing a wide range of combinations. The set of parameters which gave the least total error was selected, following equations 9, 10, and 11. For most time steps, a range of parameter values all gave the lowest error, so a single parameter value could be used for the set of four 2007 time steps. The observed-infected nodes were given 9 times more weight than the observed-healthy nodes for evaluating the final error, i.e., *α* = 0.9. Thus, the error if all nodes were predicted infected would be approximately 10%. The gravity law model was selected as the one providing minimum error.

#### Soybean rust prediction

**Figure 2:**
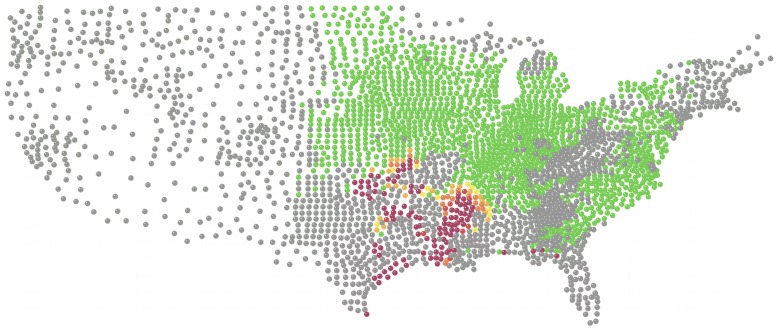
Prediction for soybean rust infection in the United States in September 2007 based on 2007 observations through August. Dark red nodes represent counties which were predicted to be infected with high probability, green nodes represent counties which were predicted to be uninfected with negligible probability of infection, and all other shades from green to dark red represent increasing probability of infection.

We applied our model to make predictions for the summer months of the years 2005 to 2008. We chose to focus on the summer months from the beginning of May to the end of September because soybean rust was less active during the other months which were not suitable for the pathogen to survive and propagate in much of the country. We used year 2007 data for construction of the model and years 2005, 2006, 2008 for validation of the model. For the one-step predictions, we used data from a first time-step to predict the next time-step, and the second time-step to predict the third and so on (Table 2). This approach is similar to what would be used when predictions are made into the near future as a support tool to help farmers decide about fungicide use depending on the prediction. For multi-step predictions, a single time-step could be used to predict many time-steps into the future, for example when predictions are needed for an entire season or year or farther into the future even if data are not available for intermediate steps. Shown in Figures 1, 2 are examples of the maps for observed rust status and the prediction for the next time step.

**Table 2:** Error percentages for different time steps for the network models considered, where 2007 time steps were used for model construction and other years were used for model validation.

Year	Time step	Multiplicative model with gravitymodel for densities	Multiplicative model with sumof densities
2005	May–June		
	June–July	2.1	2.9
	July–Aug	2.5	2.5
	Aug–Sept	3.3	4.3
2006	May–June	2.5	4.0
	June–July	3.5	4.5
	July–Aug	1.1	3.7
	Aug–Sept	2.1	5.0
2007	May–June	1.1	10.0
	June–July	1.5	14.6
	July–Aug	2.7	3.0
	Aug–Sept	4.4	4.0
2008	May–June	3.4	3.3
	June–July	2.6	2.1
	July–Aug	3.1	3.1
	Aug–Sept	0	1.2

The parameter estimates for the multiplicative model with a gravity model for source and destination densities for all the year 2007 monthly steps over summer months (i.e., May 2007 to June 2007, June 2007 to July 2007, July 2007 to August 2007, and August 2007 to September 2007) were *a_1_* = 0.01 and *a_2_* = 1. For the multiplicative model with the sum of source and destination densities they were *a_1_* = 0.01 and *a_2_* = 10.

#### Evaluation of importance of host density and wind velocity data in the model

In order to test the importance of host density and wind as model components, we randomly sampled the densities and wind values with replacement for all the counties. We compared the performance of models for the observed variables and for the randomized variables. First, the set of observed county host density values were randomly reassigned with replacement, maintaining the original observed wind speeds and directions for each county. The host densities were randomly reassigned in 500 independent simulations, and the error associated with predictions (based on the parameter estimates for the observed densities) for each simulation was recorded. The observed error was compared to the distribution of errors from the simulations. The errors from the simulations were ranked from smallest to largest and the position of the observed error within the list was noted. The rank of the observed error among the 500 values of the bootstrap distribution from randomizing host density was found to be 1 for all time-periods, indicating that the original observed host density data gave the least error. All the simulated reassignments of host densities degraded the performance. Second, the set of wind speeds and directions was randomly reassigned with replacement, maintaining the original observed host densities. The observed error was compared to the distribution of errors from 500 simulations based on the randomly reassigned wind data. The rank of the observed error among the 500 values of the bootstrap distribution from randomizing wind velocity was found to be 1 for all time-periods, which indicates that the original wind velocity data gave the least error. Again, all other simulated reassignments of wind velocities degraded the performance. Inclusion of the actual host density and wind data improved the model predictions.

### Strategic Reduction of Soybean Rust Sentinel Plot Network

We implemented the four approaches to selecting sampling nodes discussed above for the soybean rust sentinel plot network. For this data set, the number of sentinel plots sampled in each monthly transition typically varied between 400 and 600. We based the reduction in percentage plots available for each monthly transition on the number of plots with information available for that transition. We evaluated the error resulting when a reduced percentage of the original observed sentinel plot network was used for making model predictions for the 16 monthly transitions that had substantial observation numbers and higher numbers of infected nodes. For cases where sampling nodes were removed at random, we generated 50 realizations.

#### Random selection of informative nodes

The simplest way to reduce the total number of sentinel plots is to randomly sample the entire observed set of sentinel plots. We evaluated the error resulting when x% (10%, 25%, 50%, 75% and 100%) of the original observed sentinel plot network was used for making model predictions. This analysis represents a type of ‘control’ for evaluation of other methods, since it is based on, in effect, no strategy.

#### Zonal selection

In this method, we exploit the fact that disease has most commonly been found in the Southeastern US and has rarely reached the north or the west. Here, we have more nodes in the Southeast and fewer nodes in the remaining regions. This way we have a higher density of plots in regions of greater observed frequency of infection. We divided the country into three zones as follows:

1. Region1 between 25.61 and 38 degrees latitude and −98 and −67.63 degrees longitude. This is the Southeastern region with highest infection frequency.
2. Region2 between 38 and 44 degrees latitude and −110 and −98 degrees longitude.
3. Region3 between 44 and 48.77 degrees latitude and −124.15 and −110 degrees longitude.

We maintained a density of 80%, 10% and 10% of the total number of informative nodes in the network for region 1, 2 and 3, respectively.

#### Infection frequency based selection

**Figure 3:**
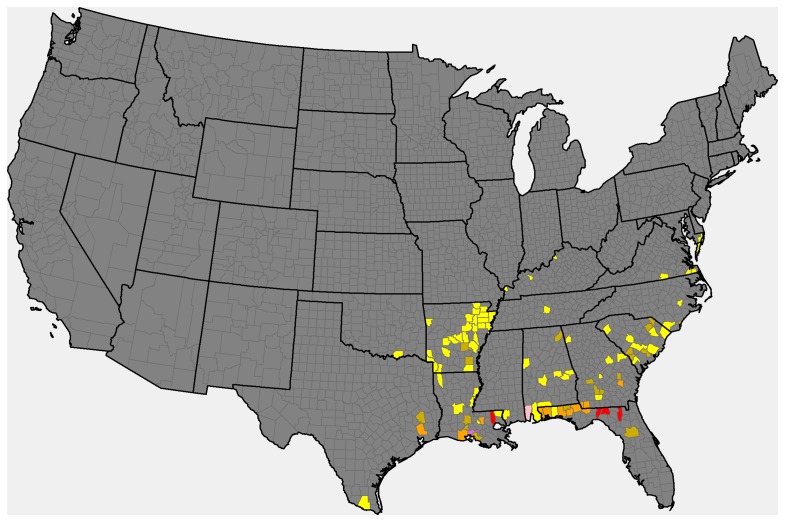
The frequency of soybean rust infection observed at each county node in the United States during our study intervals. Colors represent infection frequencies: lilac (≥10), dark pink (5–9), red (4), orange (3), gold (2), yellow (1) and grey (0).

In this method, we calculate the frequency with which each node has been observed infected (Fig. 3), and then order the nodes from highest to lowest values of frequency. The resulting network consists of nodes with non-zero frequency or frequency above a certain number.

#### Combination of past infection frequency and selection based on node strength

In this method, infection frequency and node strength were weighted in the ratio 80∶20 (after they had been scaled to be between 0 and 1) for each node, and the nodes were ordered in decreasing order of this weighted value. Only the highest x% of the whole set of nodes were considered. The rationale for this weighting was that it was still useful to emphasize counties where the epidemic was typically active, but that the ‘highly connected’ nodes should also be emphasized.

## Results

### Results for Strategic Reduction of Informative Nodes or Sentinel Plots

**Figure 4:**
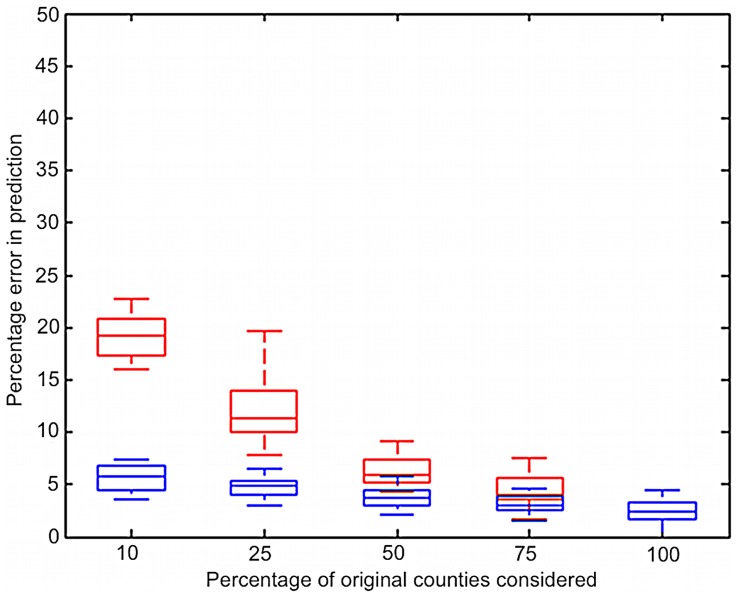
Summary of the performance of sentinel plot subsets for predicting soybean rust infection, where subsets were determined using random selection and zonal selection, over all the years. Red plots indicate results of random selection, and blue plots indicate results of zonal selection. Strategic zonal selection gives lower errors compared to random selection. Each box indicates the distribution of the means for each of the 16 time steps analyzed.

We analyzed the effects of the random sampling approach on the error in prediction using our model for reduction to 10%, 25%, 50%, and 75% of the total set (compared to 100%) and plotted the results for 50 runs for each of the 16 month-to-month transitions. There is rapid decay in performance for random sampling when the percentage sentinel plots retained goes below 50% (Fig. 4). With strategic zonal sampling, a marked improvement in the performance is achieved (Fig. 4). This summary of random selection of monitoring nodes and zonal selection strategy was constructed using the average percentage errors over 50 runs at each time step from 2005 to 2008 for both strategies.

**Figure 5:**
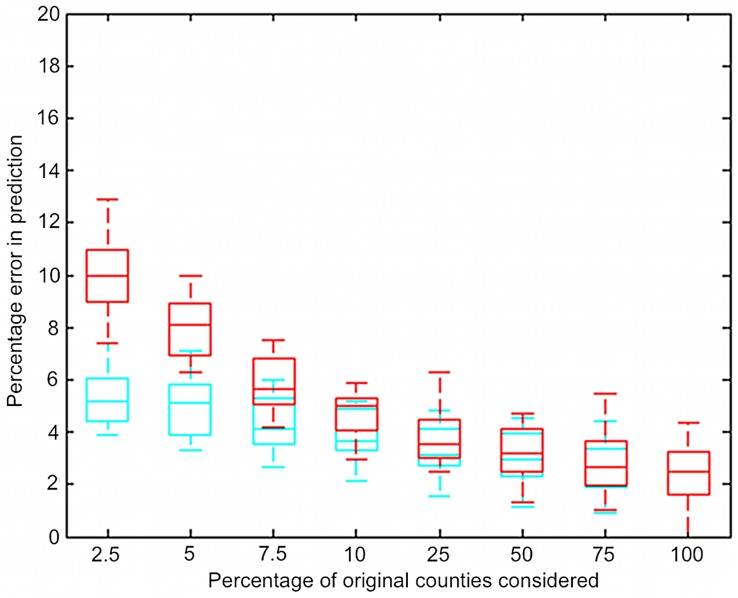
Summary of the performance of sentinel plot subsets for predicting soybean rust infection, where subsets were determined by infection frequency based selection and weighted infection frequency and node strength based selection (weighted in the ratio 80∶20), over all the years. Red plots indicate results of infection frequency based selection, and blue plots indicate results of weighted infection frequency and node strength based selection. Addition of node strength information to the infection frequencies of the nodes lowers the errors significantly. Note that the range of errors depicted is narrower than in Figure 4. Each box indicates the distribution of the means for each of the 16 time steps analyzed.

Sentinel plot subsets selected using infection frequency and combined infection frequency and node strength gave lower prediction errors. The addition of node strength information to the infection frequency information yielded lower errors in prediction (Fig. 5). The total number of nodes at each time step was about 400 to 600, hence 10% of nodes is about 40 to 60 nodes.

The summary of random selection of monitoring nodes and zonal selection strategy was constructed using the average percentage errors from all time steps from 2005 to 2008 for both strategies. For the infection frequency based selection, the frequencies varied from zero to ten. Since this strategy could not be evaluated for the exact same percentage of original nodes as the other strategy, the values were plotted by taking the percentage nearest but lower to x-axis mark-up percentages. The node property based selection strategies outperformed the random and zonal selection strategies. The addition of node strength information to the infection frequency information improved the performance substantially, with prediction errors below 5% even for when only 2.5% of the original sentinel plots were maintained (approximately 12 sentinel plots at each time step).

## Discussion

We developed and validated what is, to our knowledge, the first national-scale dynamic network model for an invasive species moving outside of human transportation networks. We compared two models for the link weights. Overall, the multiplicative model with densities multiplied as specified by the gravity law [61], [63] performed best over a wide range of time periods. The gravity model may not be the most useful in all invasive scenarios [64], so for invasive species that are markedly different than soybean rust it may be useful to start by comparing a range of model types. The multiplicative model does not allow direct analysis of the importance of the different factors involved as does an additive model, as we cannot change the weights associated with the distance, density and wind independently. But a resampling analysis demonstrated that both host density and wind speed and direction were important components of the model. The prediction errors for the model were generally low. This is in part a reflection of the fact that the disease was not observed to make substantial ‘hops’ during the observations available for modeling; that is, it was not common to have large uninfected regions dividing infected areas. This made fitting easier. (When the disease did move into the interior of the U.S., spores were rained out along the entire route of the long-distance incursions, but those spores landing in the more southern locations along the route resulted in more rapid disease progression due to warmer autumn temperatures that were more disease-conducive. Thus the disease appeared to spread along the route from south to north without leaving large gaps in its distribution (S. Isard, personal observation).) Our definition of prediction error, with a heavy weight on observed-infected nodes compared to observed-uninfected nodes, means that a few nodes incorrectly predicted to be infected will have negligible effect on the prediction error. The rationale for this choice in weights was that (1) it is more important to correctly predict infection than to correctly predict absence of infection, (2) absence of infection in a sentinel plot may occur even when infection does occur within the county represented by the sentinel plot, while infection of the sentinel plot is sufficient to prove infection within the county, and (3) the observations include a large number of sentinel plots in the north that have never been infected, so it is ‘too easy’ to correctly predict their absence of infection.

While models such as that of Wang et al. [33] consider a network to be homogeneous, our model also takes into account the weights associated with the links [34] and we used observed field data to fit these weights and test the model performance. The crop network model developed by Margosian et al. [24] considers links between adjacent counties and the effect of crop density in these counties. Our model enhances such a network model by not only considering the adjacent counties but also those that are further apart based on a flexible threshold on the distance, and we also incorporated the effect of wind speed and direction and the availability of an alternative host (kudzu) as a reservoir for the pathogen. While the model currently takes into account the effect of wind in carrying the spores from one location to another, other factors like temperature [65], moisture [66], and UV radiation [67] could also be incorporated. Incorporating spore trap data in the model as a measure of amount of inoculum present in an area could further improve the predictions. Another potential improvement, when data are available, would be information about what decisions farmers have made for disease management; if farmers to the south are commonly using fungicides to manage soybean rust, disease risk in the north may be reduced. Ultimately models of farmer decision-making in response to dynamic weather systems [68], [69], [70], [71] may be merged with network models of disease for evaluation of linked epidemic and decision-making networks [23]. A related measure, and also potentially expensive to obtain, would be information about infection severity within counties.

In the evaluation of strategies for selecting subsets of sentinel plots under resource limitations, the use of a network model metric, node strength, proved to be valuable. The strategy that combined use of past frequency of infection with node strength allowed predictions of epidemic progress with errors below 5% even when the number of sentinel plots was reduced from approximately 500 per time step to approximately 12 per time step. This information allows maintenance of prediction quality with a much smaller resource demand. Obtaining the initial information about frequency of infection is a prerequisite and is, itself, potentially expensive. However, once reliable information about frequency of infection over time is available, the additional information about node strength is relatively inexpensive to obtain through modeling.

The greatest efficiency for predictions will be obtained when models and parameter estimates can be adjusted for new systems without the requirement for parameterizing models completely *de novo*. This type of modeling approach has general application for other plant or animal epidemics or insect infestations studied across large or small landscapes [72]. The disease prediction model can potentially be applied to other wind-borne invasives with minor species-specific adaptations or modifications. For example, the new race of the wheat stem rust pathogen, Ug99, may be introduced to the US in the near future, where effective disease resistance is not common in wheat varieties [73]. This pathogen will likely exhibit a similar pattern of annual invasion, overwintering in the south, in this case further west in south Texas and northern Mexico, and moving northward in the US during the wheat growing season. (A simplifying factor for soybean rust modeling is that effective disease resistance is not yet available. For other pathogens, such as wheat rust fungi, the host landscape will be disrupted through the deployment of different disease resistance genes.) The approach for reduction of the number of monitoring sites can also potentially be generalized to wheat stem rust and other monitoring networks, by identifying those locations a priori which are most likely to be frequently infected and which are most likely to have high node strength. Future work for strategic positioning of monitoring sites may also involve sampling based on other node characteristics such as betweenness and clustering coefficients.

Soybean rust is a dramatic example of a pathogen for which agricultural and unmanaged host systems are linked, through soybean, kudzu, and other legume species [49], [50], [74]. Network models have also been used to study movement of *Phytophthora* spp. through landscapes of multiple host species [41]. Network models may also prove useful for studying large scale epidemics of other pathogens shared among host systems, such as *Barley yellow dwarf virus*, which impacts competition among California native grasses and invasive weedy grasses [75], , and *Macrophomina phaseolina*, where populations are shared among tallgrass prairie and Great Plains agricultural systems [77]. While increased host biodiversity may generally provide disease regulation as an ecosystem service, the presence of different types of hosts in landscapes may in some cases increase disease risk [78].

Evaluation of invasion networks will be an important component of climate change scenario analyses at continental scales [79], [80], [81]. Once a region is saturated with inoculum due to disease-conducive weather conditions, the effects of breaks in host connectivity may be less important for slowing disease [82]. Many small-scale forecasting models exist for reproduction of plant pathogens, arthropods, and other species as a function of weather, where these may be rescaled for use in national or continental network models [83]. The effect of host community variability on epidemics has been evaluated in many agricultural systems at smaller scales, where connectivity may be manipulated experimentally most readily within fields of mixed cultivars or species [84]. For example, the effect of disrupted host connectivity through cultivar mixtures was higher for a wheat rust than another facultative biotroph wheat pathogen in a direct experimental comparison [85].

Dynamic network models are likely to prove an important tool for integrating information from national and global monitoring systems such as the proposed US National Ecological Observatory Network program and new programs in response to potential new invasives such as wheat stem rust race Ug99. There are a number of programs for continental ecological studies such as NEON that might benefit from the example of the coordinated soybean rust sentinel plot network and analyses [46], [86], [87], [88] and from the use of network models to guide analysis of the relationships among communities in different zones of an observatory network [2]. As part of the educational and citizen science programs of national ecological studies such as NEON [89], links with farmers and extension agents who have strong interests in the analysis of invasive pathogens has potential to create new synergies. The new technologies for broad geographic analyses of remote sensing data in programs such as NEON [90], [91] may also contribute as an information source for tracking plant infection processes, especially for cases where the confidence associated with distinguishing effects of pathogens from other stressors is high.
